# Facilitating best practices in collecting anomalous scattering data for *de novo* structure solution at the ESRF Structural Biology Beamlines

**DOI:** 10.1107/S2059798316001042

**Published:** 2016-03-01

**Authors:** Daniele de Sanctis, Marcus Oscarsson, Alexander Popov, Olof Svensson, Gordon Leonard

**Affiliations:** a ESRF – The European Synchrotron, 71 Avenue des Martyrs, 38000 Grenoble, France

**Keywords:** ESRF, MAD, experimental phasing, *MXCuBE*2

## Abstract

The constant evolution of synchrotron structural biology beamlines, the viability of screening protein crystals for a wide range of heavy-atom derivatives, the advent of efficient protein labelling and the availability of automatic data-processing and structure-solution pipelines have combined to make *de novo* structure solution in macromolecular crystallography a less arduous task. Nevertheless, the collection of diffraction data of sufficient quality for experimental phasing is still a difficult and crucial step. Here, some examples of good data-collection practice for projects requiring experimental phasing are presented and recent developments at the ESRF Structural Biology beamlines that have facilitated these are illustrated.

## Abstract 2

Best practices in data collection for experimental phasing at the ESRF Structural Biology beamlines are described.

## 1. Introduction   

The constantly increasing number of macromolecular crystal structures deposited in the Protein Data Bank (PDB; Berman *et al.*, 2007 ▸), the increasing number of protein folds that these contain and the advent of more sensitive methods (McCoy *et al.*, 2007 ▸; Read, 2001 ▸; DiMaio *et al.*, 2011 ▸) has meant that molecular replacement (MR) is the overwhelming choice for structure solution in macromolecular crystallography (MX). However, the solution of the crystal structures of biological macromolecules of unknown fold and of large protein–protein or protein–nucleic acid complexes still often requires experimental phase determination. Most experiments for *de novo* structure determination routinely exploit anomalous scattering *via* the multiwavelength anomalous dispersion (MAD; Smith, 1991 ▸; Hendrickson, 1991 ▸) or single-wavelength anomalous dispersion (SAD; Rice *et al.*, 2000 ▸; Dauter, 2002 ▸; Dauter *et al.*, 2002 ▸) techniques. Such experiments are facilitated by the almost continuous evolution of tunable synchrotron beamlines at which experimenters are able to accurately measure the absorption edges of almost any anomalous scatterer that can be introduced into a crystal and to collect diffraction data at various energies around these in order to optimize anomalous and dispersive signals.

When carried out on a single cryocooled crystal, MAD and SAD experiments, in principle overcome the problems of non-isomorphism that can plague isomorphous replacement experiments. Nevertheless, in experiments where anomalous signals are small (*i.e.* S-SAD; Hendrickson & Teeter, 1981 ▸; Dauter *et al.*, 1999 ▸; Liu & Hendrickson, 2015 ▸; Weinert *et al.*, 2015 ▸) care must be taken to reduce systematic errors and, most of all, errors introduced by radiation damage. Radiation damage is exacerbated in MAD/SAD experiments because it not only results in a decrease, as a function of absorbed X-ray dose (Seltzer, 1993 ▸; Holton, 2009 ▸), in the resolution to which a crystal diffracts, it also causes specific chemical damage including disulfide-bond breakage (Weik *et al.*, 2000 ▸; Leiros *et al.*, 2001 ▸; Ennifar *et al.*, 2002 ▸), changes in electronic state (Berglund *et al.*, 2002 ▸; Schlichting *et al.*, 2000 ▸) and, very importantly, reduction in the ‘occupancy’ of anomalous scatterers (Ramagopal *et al.*, 2005 ▸; Evans *et al.*, 2003 ▸; Ravelli *et al.*, 2005 ▸). Moreover, specific radiation damage of the types mentioned above may already occur at doses much lower than the Garman Limit (Owen *et al.*, 2006 ▸). Sometimes the effects of radiation damage can be used to an experimenter’s advantage, as in radiation-damage-induced phasing (RIP; Ravelli *et al.*, 2003 ▸; de Sanctis & Nanao, 2012 ▸) or in the modelling of the reduced occupancy of heavy atoms (Schiltz *et al.*, 2004 ▸). However, there are often limitations to the use of such approaches on a systematic basis and, unless this is intentional, it is advisable to minimize radiation damage as much as possible. Here, we briefly describe data collection strategies aimed at reducing both systematic errors and radiation damage during MAD/SAD experiments and describe how such strategies can best be put into practice at the ESRF Structural Biology Beamlines.

## 2. ESRF MAD Structural Biology Beamlines   

**Figure 1:**
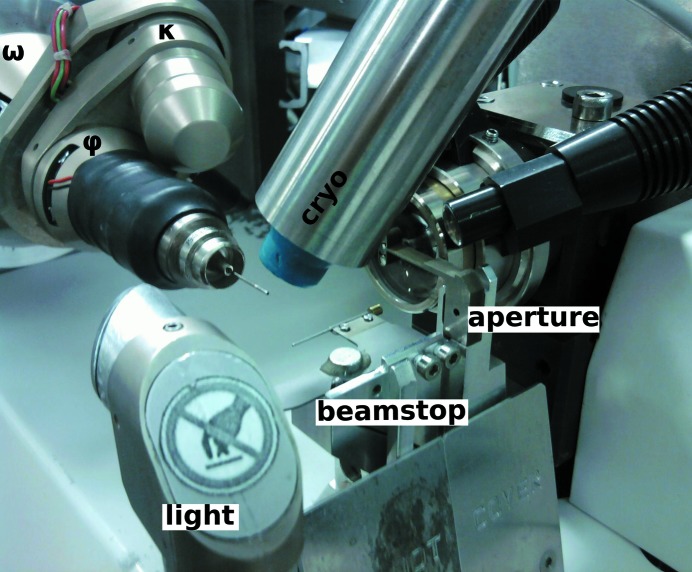
Close-up view of the sample environment on ID29 at the ESRF. Goniometer motors and main components are labelled.

The ESRF operates three tunable-wavelength endstations each covering the energy range 6–20 keV: ID23-1 (Nurizzo *et al.*, 2006 ▸), ID29 (de Sanctis *et al.*, 2012 ▸) and ID30B (Mueller-Dieckmann *et al.*, 2015 ▸). The three stations are equipped with similar diffractometers, MD2 (a microdiffractometer; Arinax, Moirans, France; Perrakis *et al.*, 1999 ▸), MD2M (a minidiffractometer; Arinax, Moirans, France) and MD2S (a microdiffractometer for screening; Arinax, Moirans, France), all with on-axis sample visualization. ID23-1 and ID29 have a fixed focal spot size at the sample position and are equipped with beam-defining apertures which allow tailoring of the X-ray beam size to a minimum dimension of 10 µm in diameter (Fig. 1 ▸). ID30B offers a variable beamsize at the sample position ranging from 20 to 200 µm. All three endstations share a similar data-collection geometry (ω axis for oscillation scans horizontal and perpendicular to the X-ray beam) and are equipped with MK3 minikappa goniometers (Brockhauser *et al.*, 2013 ▸) for crystal realignment and fluorescence detectors for the measurement of absorption-edge scans, which are carried out at the click of a button and are automatically analysed with *CHOOCH* (Evans & Pettifer, 2001 ▸) for the determination of anomalous scattering factors around the absorption edge end and of ‘peak’ and ‘inflection-point’ energies.

## 3. The *MXCuBE*2 concept   

**Figure 2:**
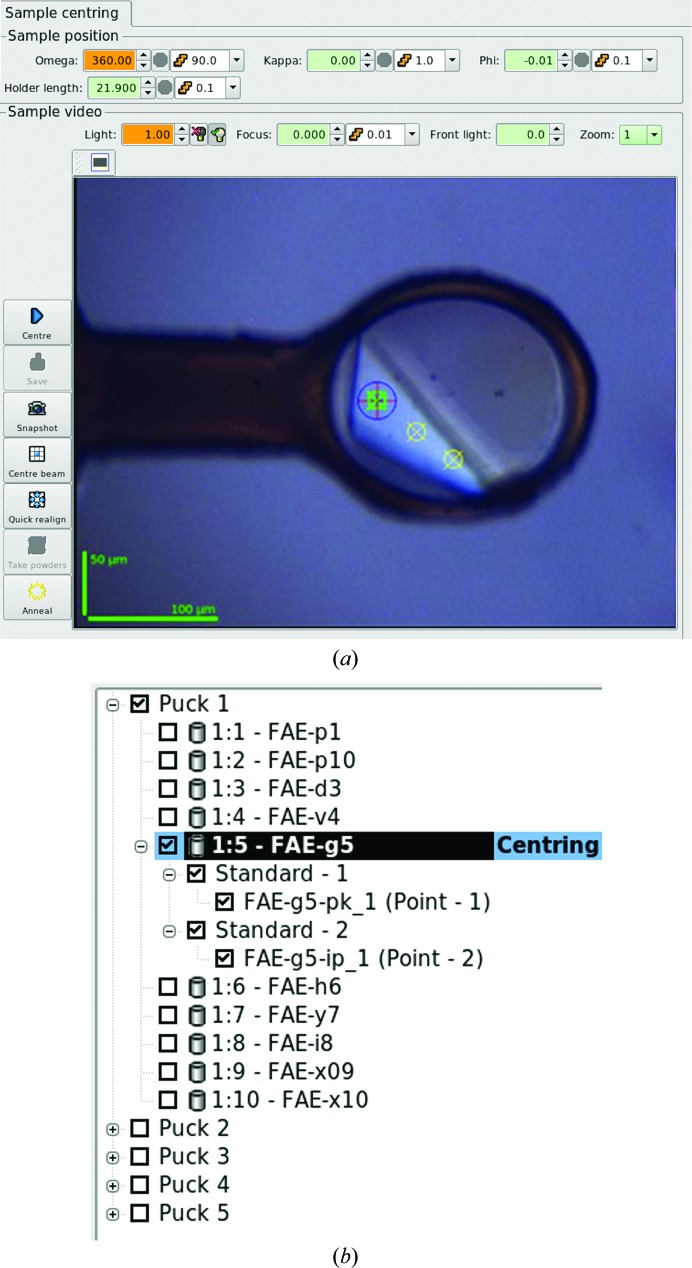
(*a*) Detail of the *MXCuBE*2 interface. The active centred position is marked in lime and other saved positions are marked in yellow. To activate a saved position it is sufficient to click on it and multiple positions can be simultaneously activated. (*b*) Detail of the queue of *MXCuBE*2. In the example shown a two-wavelength MAD (peak and inflection) data set is to be collected, with each data set collected from a different saved position of the sample 1:5 FAE-g5.

User control of experiments on ID23-1, ID29 and ID30B is, as for all of the ESRF facilities for MX, *via* the *MXCuBE*2 (http://www.esrf.eu/mxcube2 and https://github.com/mxcube) graphical user interface (GUI), the full functionality of which will be presented elsewhere (Oscarsson *et al.*, in preparation). Briefly, *MXCuBE*2, the successor to *MXCuBE* (Gabadinho *et al.*, 2010 ▸), has been designed to simplify the planning and execution of MX experiments *via* a single, integrated and intuitive interface, which facilitates the carrying out of experiments with elaborate data-collection strategies by minimizing error-prone iterative manual intervention. Two main aspects of *MXCuBE*2 are relevant for experiments exploiting anomalous scattering in *de novo* crystal structure determination: firstly, *MXCuBE*2 relies on the concept of ‘saved positions’ for data collection from any given sample (Fig. 2 ▸). Each saved position can be assigned a series of data collections to build up any complex data-collection scheme. Secondly, the *MXCuBE*2 data-collection queue can be prefilled by expert experimental descriptors, such as *EDNA* (Incardona *et al.*, 2009 ▸) and workflows (Brockhauser *et al.*, 2012 ▸) that automate data collection and analysis.

## 4. Good data-collection practice as implemented in *MXCuBE*2   

### 4.1. Calculation of data-collection strategies   

**Figure 3:**
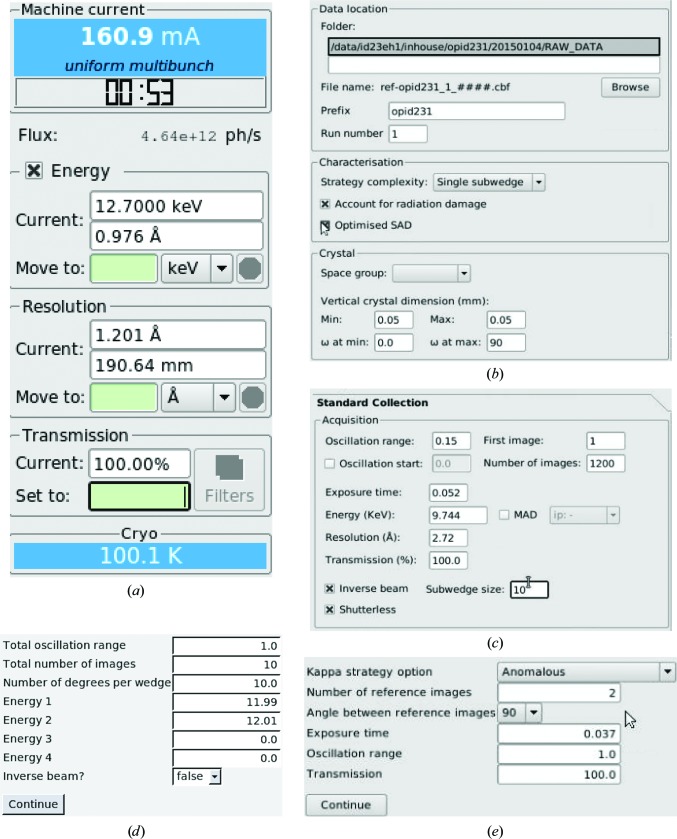
(*a*) *MXCuBE*2 displays the photon flux incident on the sample in photons per second. The value is adjusted when changing the beam-defining aperture. This value and the beam size are given to *EDNA* to allow an estimation of the dose rate deposited on the crystal when calculating data-collection strategies. (*b*) Panel for *EDNA* characterization. Besides information on the data path and file name, users can adjust the complexity of the data collection (as in *BEST*; Bourenkov & Popov, 2010 ▸), request a specific strategy for anomalous data collection by opting for ‘Optimize SAD’ (Bourenkov & Popov, 2010 ▸) and specify the crystal dimensions and space group, if already known. (*c*) Inverse beam is provided as an additional option for ‘standard data collection’, in which the number of frames composing each subwedge (in this case ten) for the two data sets to be collected is defined. (*d*) Interleaved MAD workflow interface: up to four energies can be interleaved and frames can optionally be recorded in inverse-beam geometry. (*e*) Workflow interface for κ-angle reorientation: the anomalous strategy aims to align an evenfold axis parallel to ω. Possible κ strategies are described in Brockhauser *et al.* (2013 ▸).

Calculation of a suitable data-collection strategy is among the ‘good practices’ handed down by crystallographers (Dauter, 1999 ▸; Flot *et al.*, 2006 ▸). Typically, the goal is to determine the minimal angular range and a convenient oscillation range (slicing) and to ensure that complete data with the desired multiplicity are collected. To ensure the calculation of optimal data-collection strategies at ESRF MX beamlines, the *EDNA* pipeline (Incardona *et al.*, 2009 ▸) is used *via* the *MXCuBE*2 ‘Characterization’ tab. The successor to *DNA* (Leslie *et al.*, 2002 ▸), *EDNA* has been developed to take advantage of the evolution of software such as *LABELIT* (Sauter *et al.*, 2004 ▸), *BEST* (Bourenkov & Popov, 2010 ▸) and *RADDOSE* (Paithankar & Garman, 2010 ▸) to produce data collection strategies which take into account global radiation damage. Crystal symmetry is determined from two or four diffraction images using either *LABELIT* or *MOSFLM*; X-ray dose is calculated in *RADDOSE*, assuming an ‘average’ crystal composition, using the energy of the incident beam, photon flux and beam size, which are automatically provided by *MXCuBE*2, and the dimensions of the crystal under study (Fig. 3 ▸). The dose is subsequently used in *BEST* to propose a diffraction plan, which is added, in the form of a new data collection, to the *MXCuBE*2 queue (Fig. 2 ▸). Crystal size can be measured directly from the sample view using a dedicated tool that converts lengths measured in pixels to micrometres. Users can also specify their requirements for data collection, such as preferred angular range or desired multiplicity, request an ‘anomalous strategy’ (Bourenkov & Popov, 2010 ▸; Fig. 3 ▸ *b*) and, if necessary, modify the parameters of the proposed strategy. For experiments aimed at *de novo* structure solution, the collection of highly redundant data is often advisable, and this becomes even more relevant when weak anomalous signals are to be exploited (Dauter & Adamiak, 2001 ▸; Cianci *et al.*, 2008 ▸; Akey *et al.*, 2014 ▸; Weinert *et al.*, 2015 ▸; Liu & Hendrickson, 2015 ▸).

### 4.2. Crystal reorientation   

**Figure 4:**
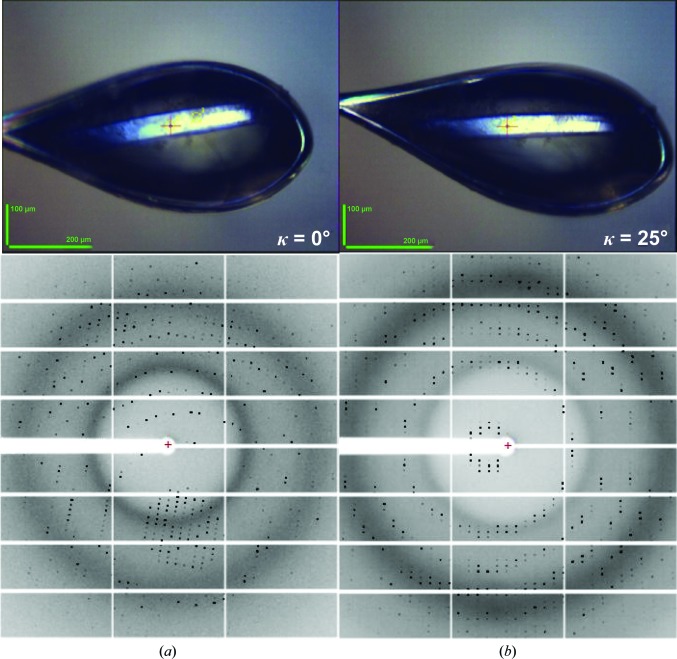
Effect of the alignment of a rod-like crystal along one of its evenfold symmetry axes, starting from (*a*) a random orientation, the result of the harvesting from the crystallization drop, to (*b*) a final reorientation with, in this case, c* parallel to ω.

Most MX beamlines at third-generation synchrotron sources are equipped with a single-axis goniometer to achieve high mechanical stability (Fig. 1 ▸), a requirement that has become particularly relevant since the advent of microfocus and microbeam beamlines (Flot *et al.*, 2010 ▸; de Sanctis *et al.*, 2012 ▸). However, when exploiting anomalous scattering in MX the use of a single-axis goniometer presents clear limitations for experiments designed to reduce systematic errors by either measuring the ‘true multiplicity’ (*i.e.* the multiplicity obtained by recording reflections from multiple different crystal orientations) or orienting a specific unit-cell axis parallel to the ω rotation axis so that Friedel mates can be measured on the same diffraction image. To overcome this limitation, ESRF tuneable beamlines are equipped with MK3 minikappa gonio­meters (Brockhauser *et al.*, 2013 ▸) that allow crystal reorientation, while sample rotation is performed around ω (see Fig. 1 in Brock­hauser *et al.*, 2013 ▸). Truly redundant data can straightforwardly be obtained by combining data sets collected from the same crystal at different κ angles. In this way, equivalent reflections are recorded on a different area of the two-dimensional detector and with different X-ray path lengths through the sample. *MXCuBE*2 facilitates such experiments by allowing the creation of a queue of data collections, from the same or from different positions in a crystal, at different, user-defined κ angles. A more complicated use of the functionality of the MK3 consists of reorienting the crystal so that an evenfold rotation axis of the unit-cell point group is oriented parallel to the ω rotation axis (Fig. 4 ▸). This allows the collection of both reflections in a Friedel pair on the same diffraction image, thus ensuring that they are measured at the same time and after the crystal has suffered the same X-ray dose. Calculating the κ and φ angles required for such a reorientation can be a complicated task that is beyond the scope of even experienced experimenters, as it requires knowledge of the beamline hardware configurations and limits. However, this procedure has been automated in the ‘kappa reorientation’ workflow (Brock­hauser *et al.*, 2012 ▸) directly interfaced with *MXCuBE*2. In this workflow, two 1° oscillation images collected 90° apart in ω at κ = 0° are used to identify the crystal Bravais lattice and calculate a crystal orientation matrix in the laboratory frame. Subsequently, the *STAC* server (Brock­hauser *et al.*, 2013 ▸) calculates the κ and φ angles that align an evenfold axis parallel to ω. Once the crystal has been reoriented, the user centres the sample in the X-ray beam, a data-collection strategy for the optimum collection of anomalous data is then calculated with *EDNA* and, upon the approval of the experimenter, executed. In Table 1 ▸ we report data-collection statistics from a crystal of the selenomethionine-derivative of the feruloyl esterase module of xylanase 10B from *Clostridium thermocellum* (Prates *et al.*, 2001 ▸; PDB entry 1gkk) obtained in space group *P*4_2_2_1_2. Two data sets were collected from different positions of a single crystal. The first data set was collected with the crystal in a random orientation, as harvested in the nylon loop, and the second with the c* axis aligned parallel to ω. In both cases the data-collection strategies were as recommended by *EDNA*/*BEST*. As can be seen, reorientation along the c* axis results in a larger anomalous signal (‘DelAnom’ and ‘Mid-Slope of Anom. Normal Probability’ in Table 1 ▸), despite very similar total doses being used to collect data sets with very similar completeness and multiplicity.

### 4.3. Inverse-beam data collection   

**Figure 5:**
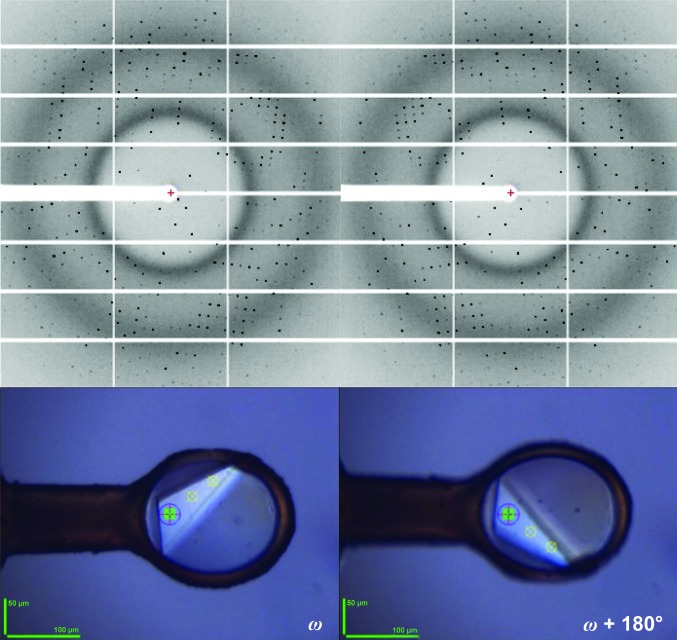
Inverse-beam data collection allows the measurement of reflections in a Friedel pair close together in time by alternately collecting diffraction images at ω (left) and ω + 180° (right).

Although the MK3 is an extremely useful device for aligning crystals during MAD/SAD experiments (see above) or for aligning crystals such that a long unit axis is parallel to ω, a combination of unfortunate crystal orientation inside the sample loop and the limited (24°) α opening angle of the MK3 may mean that the reorientation required is out of reach. In such cases it may well be desirable in MAD/SAD experiments to ensure the collection of both reflections in a Friedel pair close together in time and in X-ray dose by resorting to inverse-beam geometry (Hendrickson *et al.*, 1985 ▸; Dauter, 1997 ▸; Fig. 5 ▸). In an inverse-beam experiment the two reflections in a Freidel pair are collected at rotation angles ω and ω + 180°, respectively, and an inverse-beam geometry experiment consists of subdividing the total angular range to be collected into two data sets 180° apart in ω and collecting them, often broken down into ‘subwedges’, in sequence (Figs. 3 ▸ *c* and 5 ▸). The beamline-control software takes care of the correct starting angles, image and run numbering for each data set. The two data sets sets are processed separately and then scaled together to provide the final data set. Although, to our knowledge a systematic study on the beneficial use of inverse-beam geometry is not available, many examples of successful phasing experiments using this technique have been reported in the literature (Liu *et al.*, 2012 ▸, 2013 ▸; Akey *et al.*, 2014 ▸).

### 4.4. Interleaved MAD data collection   

In the early days, MAD experiments were complicated and required intervention from the local staff to properly prepare the beamline (*i.e.* change the energy and realign the beamline optical components and sample environment) at each wavelength at which MAD data were collected. The degree of automation achieved during the last decade at the ESRF MX beamlines (Arzt *et al.*, 2005 ▸) and at other synchrotrons worldwide (Soltis *et al.*, 2008 ▸; Stepanov *et al.*, 2011 ▸; Cork *et al.*, 2006 ▸) has simplified and stabilized the operation of tunable MX beamlines to the extent that energy changes during MAD experiments are now almost transparent to the user. This increase in user-friendliness has allowed users to concentrate on the optimization of experiment design, which is particularly relevant when extracting small anomalous signals for *de novo* structure solution or when samples are sensitive to radiation damage. As already noted, *MXCuBE*2 is designed to allow the modular assembly of data-collection protocols and gives the opportunity to build the experiment sequences. One such experiment sequence available as a workflow in *MXCuBE*2 and in which subwedges of complete diffraction data sets are alternately collected at different energies is ‘interleaved MAD’ (Finke *et al.*, 2016 ▸), a protocol which ensures that the dispersive differences between the same reflections in different data sets are minimally affected by X-ray damage. To demonstrate the ease of carrying out such a data-collection protocol, we collected, from two different positions of the same crystal of the feruloyl esterase module of xylanase 10B from *C. thermocellum* (Prates *et al.*, 2001 ▸) obtained in space group *P*2_1_2_1_2_1_, a ‘classic’ two-wavelength MAD data set and a MAD data set in which the collection of data at the two wavelengths was interleaved by 10° subwedges. The resulting data processing statistics are reported in Table 2 ▸ and show the individual data sets to be of similar quality. The interface for the ‘interleaved MAD’ workflow permits interleaving of the collection of MAD diffraction data at up to four different energies and, furthermore, can be used in combination with inverse-beam geometry (Fig. 3 ▸ *d*). Evolution of the workflow to provide new interleaving protocols is straightforward and can easily be implemented. For example, interleaving of the collection of diffraction data at different detector distances or κ angles, for example, which should reduce systematic detector errors or compensate for suboptimal absorption correction at lower energy, is accessible by just building the desired data-collection sequence in the *MXCuBE*2 GUI.

### 4.5. Multi-positional data collection   

The advent of microfocus (Flot *et al.*, 2010 ▸) and microbeam (de Sanctis *et al.*, 2012 ▸) endstations dedicated to MX allows the measurement of diffraction data from smaller samples and maximizes signal-to-noise ratios by matching beam and crystal sizes or the scanning of a larger crystal to find its best diffracting area (Sanishvili *et al.*, 2008 ▸; Bowler *et al.*, 2010 ▸). Microbeams also allow the collection of diffraction data from different parts of crystals larger than the X-ray beam. *MXCuBE*2 allows users to centre different positions of large crystals in the X-ray beam, to save and store these positions and to link each position with a series of data collections (Fig. 2 ▸). In this way, it is possible to sequentially collect multiple data sets from the same sample. This functionality enables different possible strategies: by collecting complete data sets at different positions it is possible to increase multiplicity while escaping radiation damage; for highly radiation-sensitive samples partial data sets can be collected at each position and merged to produce a single complete data set to higher resolution than might otherwise have been the case; in MAD experiments data sets at different energies can be collected from different positions of the same crystal. This data-collection method is fully exploited in the *MeshAndCollect* data-collection workflow (Zander *et al.*, 2015 ▸), in which the positions of micrometre-sized crystals are identified by diffraction and automatically saved and a partial data set is collected at each centred point. While *MeshAndCollect* completely automates the procedure, beamline users can optically select the centring positions and define a data collection plan for each of them. The same rationale can be applied when collecting data sets at different wavelengths to perform MAD/SAD phasing experiments. In fact, although SAD phasing has become extremely popular (also thanks to the improvement in phasing and density-modification software), the fast performance of pixel detectors and beamline automation nowadays allows a second data set to rapidly be collected at another energy to obtain experimental phases of much higher quality (González, 2003 ▸). In order to avoid contamination with radiation damage in the second (or a third or subsequent) data set, it would be advisable to collect each data set from an undamaged volume (Fig. 2 ▸ *b*). A caveat when applying such strategies is that the diffraction quality over the length and/or area of a crystal can often be heterogeneous (Sanishvili *et al.*, 2008 ▸; Bowler *et al.*, 2010 ▸); the recommendation would then be to characterize each position (using *EDNA*/*BEST*) to make sure that the crystal quality is uniform. For the reason above, although the execution of helical (Flot *et al.*, 2010 ▸) or vector (Pothineni *et al.*, 2014 ▸) data collections is routinely available in *MXCuBE*2, these must be carefully pondered before they are carried out.

## 5. Conclusions   

Experimental phasing by anomalous dispersion techniques are the most successful and common *ab initio* methods for structure determination. The success of an anomalous dispersion experiment strongly relies on the data quality, and this becomes more significant when small anomalous signals are expected. In such circumstances, experiment design and the tools to perform experiments play a major role. Here, we have presented a list of the most common ‘best practices’ to be used in the collection of high-quality diffraction data for use in MAD or SAD experiments. *MXCuBE*2, the experiment-control GUI available at the ESRF Structural Biology Beamlines, facilitates these by a number of different means. *MXCuBE*2 allows the use of *EDNA*, *RADDOSE* and *BEST* to propose data-collection strategies that take into account crystal decay caused by radiation damage, it allows the straightforward implementation of complex data-collection protocols such as inverse-beam and interleaved data-collection experiments and it allows the efficient use of minikappa goniometers and of multi-position and multi-crystal data collections. Moreover, the *MXCuBE*2 data-collection queue modularity smooths the way for the integration of novel data-collection methods and data analysis, as already demonstrated with the *MeshAndCollect* pipeline. *MXCuBE*2 is constantly evolving to incorporate new experiment types and to hand them over to the MX user community.

## Figures and Tables

**Table 1:** Data-collection and processing statistics for data sets collected from a random orientation and with c* aligned with the rotation axis using the MK3 device Data were cut at 3.5 Å resolution to properly compare the anomalous signal in the two data sets. No indication of global radiation damage, such as an increase in the unit-cell volume or in the overall *B* factor, is observed in the data. Values in parentheses are for the outer shell.

	Random	κ (c*)
Beamline	ID29	ID29
Wavelength (Å)	0.9791	0.9791
Temperature (K)	100	100
Detector	Pilatus3 6M	Pilatus3 6M
Data-collection time (s)	50	44
Photon flux (photons s^−1^)	3.5 × 10^11^	4.5 × 10^11^
Crystal-to-detector distance (mm)	429.2	463.1
Rotation range per image (°)	0.05	0.1
Total rotation range (°)	80	80
Space group	*P*4_1_2_1_2	*P*4_1_2_1_2
Unit-cell parameters (Å)	*a* = *b* = 111.38, *c* = 65.40	*a* = *b* = 111.58, *c* =65.59
Mosaicity (°)	0.060	0.064
Resolution range (Å)	40–3.5 (3.83–3.50)	40–3.5 (3.83–3.50)
Total No. of reflections	30994 (7365)	30586 (7637)
No. of unique reflections	5505 (1291)	5500 (1285)
Completeness (%)	99.5 (100)	98.6 (97.9)
Multiplicity	5.6 (5.7)	5.4 (5.5)
〈*I*/σ(*I*)〉	36.8 (26.1)	49.4 (38.4)
*R*_r.i.m._ †	0.040 (0.064)	0.032 (0.039)
ISa	31.04	31.52
DelAnom	0.771 (0.448)	0.852 (0.804)
Mid-Slope of Anom. Normal Probability	2.564	3.936

† *R*_r.i.m._ = 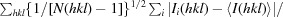 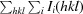.

**Table 2:** Data-collection parameters and processing statistics for the feruloyl esterase module of xylanase 10B from *C. thermocellum* (Prates *et al.*, 2001 ▸; PDB entry 1gkk) Data sets were collected from a *P*2_1_2_1_2_1_ crystal in the form of a ‘classic’ two-wavelength MAD experiment and a ‘interleaved’ MAD experiment, in which data are collected in 10° subwedges alternating between the two energies. Data were cut at 3.5 Å resolution to properly compare the anomalous signal in the two data sets. No indication of global radiation damage, such as an increase in the unit-cell volume or in the overall *B* factor, is observed in the collected data. Values in parentheses are for the outer shell.

	Peak ‘classic’	Inflection ‘classic’	Peak ‘interleaved’	Inflection ‘interleaved’
Beamline	ID29	ID29	ID29	ID29
Wavelength (Å)	0.9791	0.9793	0.9791	0.9793
Temperature (K)	100	100	100	100
Detector	Pilatus3 6M	Pilatus3 6M	Pilatus3 6M	Pilatus3 6M
Data-collection time (s)	24	24	24	24
Photon flux (photons s^−1^)	1.5 × 10^11^	1.5 × 10^11^	1.5 × 10^11^	1.5 × 10^11^
Crystal-to-detector distance (mm)	391.77	391.75	391.73	391.56
Rotation range per image (°)	0.1	0.1	0.1	0.1
Total rotation range (°)	120	120	120	120
Space group	*P*2_1_2_1_2_1_	*P*2_1_2_1_2_1_	*P*2_1_2_1_2_1_	*P*2_1_2_1_2_1_
Unit-cell parameters (Å)	*a* = 64.82, *b* = 108.42, *c* = 113.21	*a* = 64.86, *b* = 108.50, *c* = 113.29	*a* = 64.80, *b* = 108.42, *c* = 113.16	*a* = 64.80, *b* = 108.40, *c* = 113.17
Mosaicity (°)	0.039	0.040	0.042	0.043
Resolution range (Å)	40–3.5 (3.83–3.50)	40–3.5 (3.83–3.50)	40–3.5 (3.83–3.50)	40–3.5 (3.83–3.50)
Total No. of reflections	44499 (10699)	44430 (10671)	44492 (10384)	44418 (10620)
No. of unique reflections	10397 (2478)	10367 (2472)	10384 (2465)	10359 (2470)
Completeness (%)	98.9 (99.5)	98.7 (99.3)	98.9 (99.3)	98.8 (99.5)
Multiplicity	4.3 (4.3)	4.3 (4.3)	4.3 (4.3)	4.3 (4.3)
〈*I*/σ(*I*)〉	28.5 (27.1)	28.8 (26.9)	31.1 (28.9)	30.2 (27.9)
*R*_r.i.m._ †	0.046 (0.044)	0.045 (0.044)	0.042 (0.042)	0.043 (0.043)
ISa	21.82	22.50	25.62	25.06
DelAnom	0.679 (0.678)	0.696 (0.674)	0.730 (0.679)	0.718 (0.661)
Mid-Slope of Anom. Normal Probability	2.610	2.515	2.733	2.693

† *R*_r.i.m._ = 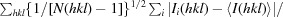 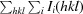.
